# Placenta mediates the effect of maternal hypertension polygenic score on offspring birth weight: a study of birth cohort with fetal growth velocity data

**DOI:** 10.1186/s12916-021-02131-0

**Published:** 2021-11-04

**Authors:** Noriko Sato, Ayako Fudono, Chihiro Imai, Hidemi Takimoto, Iori Tarui, Tomoko Aoyama, Satoshi Yago, Motoko Okamitsu, Shuki Mizutani, Naoyuki Miyasaka

**Affiliations:** 1 Department of Molecular Epidemiology, Medical Research Institute, Tokyo Medical and Dental University (TMDU), 1-5-45, Yushima, Bunkyo-ku, Tokyo, 113-8510 Japan; 2 Institute of Advanced Biomedical Engineering and Science, The Public Health Research Foundation, Tokyo, Japan; 3 Comprehensive Reproductive Medicine, Graduate School of Medical and Dental Sciences, Tokyo Medical and Dental University (TMDU), Tokyo, Japan; 4 Department of Nutritional Epidemiology, National Institute of Health and Nutrition, Tokyo, Japan; 5 Child and Family Nursing, Graduate School of Health Care Sciences, Tokyo Medical and Dental University (TMDU), Tokyo, Japan

**Keywords:** Intrauterine programming, Placenta, Blood pressure, Vascular genes, Developmental Origin of Health and Disease (DOHaD)

## Abstract

**Background:**

Low birth weight (LBW) and fetal growth restriction are associated with the development of cardio-metabolic diseases later in life. A recent Mendelian randomization study concluded that the susceptibility of LBW infants to develop hypertension during adulthood is due to the inheritance of hypertension genes from the mother and not to an unfavorable intrauterine environment. Therein, a negative linear association has been assumed between genetically estimated maternal blood pressure (BP) and birth weight, while the observed relationship between maternal BP and birth weight is substantially different from that assumption. As many hypertension genes are likely involved in vasculature development and function, we hypothesized that BP-increasing genetic variants could affect birth weight by reducing the growth of the placenta, a highly vascular organ, without overtly elevating the maternal BP.

**Methods:**

Using a birth cohort in the Japanese population possessing time-series fetal growth velocity data as a target and a GWAS summary statistics of BioBank Japan as a base data, we performed polygenic score (PGS) analyses for systolic BP (SBP), diastolic BP, mean arterial pressure, and pulse pressure. A causal mediation analysis was performed to assess the meditation effect of placental weight on birth weight reduced by maternal BP-increasing PGS. Maternal genetic risk score constituted of only “vasculature-related” BP single nucleotide polymorphisms (SNPs) was constructed to examine the involvement of vascular genes in the mediation effect of placental weight. We identified gestational week in which maternal SBP-increasing PGS significantly decreased fetal growth velocity.

**Results:**

We observed that maternal SBP-increasing PGS was negatively associated with offspring birth weight. A causal mediation analysis revealed that a large proportion of the total maternal PGS effect on birth weight was mediated by placental weight. The placental mediation effect was remarkable when genetic risk score was constituted of “vasculature-related” BP SNPs. The inverse association between maternal SBP PGS and fetal growth velocity only became apparent in late gestation.

**Conclusions:**

Our study suggests that maternal hypertension genes are strongly associated with placental growth and that fetal growth inhibition is induced through the intrauterine environment established by the placenta.

**Supplementary Information:**

The online version contains supplementary material available at 10.1186/s12916-021-02131-0.

## Background

The increasing prevalence of cardio-metabolic diseases leading to premature death is a raising concern [1]. Although major risk factors are lifestyle factors after birth, epidemiological studies have associated low birth weight (LBW) and/or fetal growth restriction (FGR) with hypertension, cardiovascular diseases, and type 2 diabetes, indicating the existence of prenatal risk factors [2–4]. However, the underlying mechanisms explaining the relationship between LBW/FGR and diseases in adulthood are not fully understood. It is suggested that maternal undernutrition and/or poor placental function could alter long-lasting body function and physiology, predisposing individuals to cardio-metabolic diseases [5, 6]. To analyze the effect of such intrauterine environment on fetal growth, the maternal genotype has been used as a proxy for the intrauterine environment [7, 8]. Maternal genetic factors may influence fetal growth directly through the alleles inherited by the fetus, or indirectly through the intrauterine environment, and this idea has been developed into the Mendelian Randomization (MR) approach in the framework of causal inference [7–10].

Recent MR studies have reported that maternal systolic blood pressure (SBP)-increasing polygenic score (PGS) causes lower birth weight through elevated maternal SBP, independently of direct fetal genetic effects [8, 10]. However, the genetic estimate of the association between maternal SBP and birth weight was in the opposite direction to the observational estimate [8]. In addition, hypertension usually develops in late life. Therefore, it may not be appropriate to apply to pregnant women the estimates of lifelong BP risks in the MR approach. Thus, it remains unclear why higher maternal SBP-increasing PGS results in lower birth weight.

FGR pathophysiology and etiology are complex, but most cases are thought to arise from placental dysfunction [4, 11]. Generally, placental growth precedes fetal growth, with the placental growth rate decreasing from about 30 weeks, and the fetus/placenta growth ratio gradually increasing until 37–38 weeks [12]. Nearly 80% of FGR are late-onset (≥ 32 weeks) [13]. In addition, it has been highlighted that placental weight is an important determinant of fetal growth in the third trimester [14], and birth and placental weight highly correlate [14, 15]. The placenta is a vascular organ, and recent GWAS uncovered numerous BP-associated genes involved in regulating the development and function of the vascular system [16–18]. Therefore, we hypothesized that BP-increasing maternal SNPs influence placental growth, which in turn affects birth weight.

Since the mediating role of the placenta was never been examined, we investigated whether maternal SBP-increasing PGS is inversely associated with placental weight and whether the placental weight mediates the effect of maternal SBP PGS on birth weight. Furthermore, by constructing a genetic BP-increasing risk score composed exclusively of vasculature-related SNPs, we aimed to clarify that the role of placental weight in the effects of vasculature-related BP SNPs on birth weight. We also aimed to confirm by haplotype analysis that maternal transmitted and non-transmitted BP-increasing alleles, but not paternal transmitted alleles, are inversely associated with birth and placental weight. Lowering of birth weight can be caused by deceleration of fetal growth velocity during pregnancy. Motivated to understand the indirect effect of maternal SBP PGS on fetal growth velocity, reflecting the fact that placental growth precedes fetal growth, we aimed to determine that maternal SBP PGS significantly decreases fetal growth velocity at the very end of pregnancy. Thus, our study uncovered that maternal genetic risk of hypertension inhibits fetal growth through the intrauterine environment established by the placenta.

## Methods

### Study cohorts

The characteristics of the two cohorts analyzed in this study are presented in Additional file 1 : Table S1. More details about the participants of these cohorts have been described previously [19–21]. The study population was comparable with the general Japanese pregnant women on perinatal parameters such as birth weight, maternal age, height, weight, body mass index (BMI), and BP [22, 23]. Chronic hypertension and hypertensive disorder of pregnancy (HDP) were excluded from this study because a previous study has shown that the effect of maternal SBP genetic score on birth weight is the same with and without HDP and hypertension [8]. Also, their frequency was low.

#### BC-GENIST

Clinical information and genotype data were obtained from the Birth Cohort Gene and ENvironment Interaction Study of Tokyo Medical and Dental University (TMDU) (BC-GENIST) project, which is a prospective mother-offspring cohort designed to evaluate the effects of prenatal environment and genotype on the epigenetic state of mothers and their offspring [19]. During the years 2015–2019, pregnant women in their first trimester were recruited to BC-GENIST at the TMDU hospital in an urban area of Japan, Tokyo (*n* = 126). All the participants provided written informed consent. Parents/guardians provided consent for their children to participate. Ninety-three mother-child pairs of Japanese ancestry, whose genotype information were available, were included in this study. There was neither HDP nor chronic hypertension.

#### TMDU pregnant women cohort

Clinical information including maternal BP and birth outcome were obtained from the retrospective cohort of women who gave birth at TMDU Hospital from 2013 to 2017 [20, 21]. The eligible criteria for this study were as follows: Japanese singleton delivery with available placental weight and live birth, without malformation, completing more than 34 weeks of gestation at the time of delivery. Maternal age was above 20 years and existing maternal BP data in two gestational periods (before 20 weeks and from 20 to 34 weeks) were available. In this study, three participants with HDP and nine with chronic hypertension were excluded. BC-GENIST participants were excluded. Total 993 women were eligible for this study. Informed consent was obtained in the form of opt-out on the website of TMDU Life Science and Bioethics Research Center.

This study was approved by the Institutional Review Board of TMDU and was conducted according to the Declaration of Helsinki.

### Observational maternal BP and perinatal outcomes

The relationship between observational BP and birth weight was analyzed using the TMDU pregnant women cohort. BP was measured at prenatal check-ups scheduled every 4 weeks until 24 weeks, every 2 weeks between 24 and 36 weeks, and weekly beyond 36 weeks of gestation. Pulse pressure (PP) was calculated by subtracting the diastolic blood pressure (DBP) from the systolic blood pressure (SBP). The PP was divided by three and added to the DBP to calculate the mean arterial pressure (MAP) [24].

Hypertension of pregnancy is classified according to the time of onset, which is also thought to be different in its pathogenesis [25]. Chronic hypertension is defined if hypertension occurs before 20 weeks and early- and late-onset HDP if HDP occurs between 20 and 34 weeks and after 34 weeks, respectively. Therefore, we divided the gestation period into three categories: [1] early, gestational age < 20 weeks [2]; middle, 20 ≤ gestational age < 34 weeks; and [3] late, 34 weeks ≤ gestational age. For each pregnant woman, the mean value of SBP, DBP, MAP, and PP at each period was used for analysis.

Birth weight and placental weight were adjusted for gestational age, fetal sex, and parity using a reference chart [12, 26]. Estimated fetal growth velocity was calculated as described previously [20]. The relatively high frequency of routine ultrasound scans at prenatal checkups in Japan allowed to estimate the weekly values of estimated fetal weight (EFW) by smoothing splines interpolation using a generalized additive model. The weekly fetal growth velocity was computed as the difference between two consecutive weekly EFW values. To compare the genetic effect size across all the phenotypes using the BC-GENIST cohort, we scaled trait to have zero mean and unit variance by *Z* score transformation.

### Genotyping and imputation of target data for polygenic score (PGS) analysis

The DNA was extracted from maternal peripheral blood and cord blood of the BC-GENIST participants using a standard procedure [19]. All samples were genotyped with the Illumina Infinium Asian Screening Array BeadChip by iScan, in accordance with the Illumina protocol, at the Center for Molecular Biology and Genetics/Core-Lab, Graduate School of Regional Innovation Studies, Mie University, Mie, Japan. Genotype calling was performed for all samples as a single project using the Genotyping Module (version 1.9) of the GenomeStudio data analysis software package. The quality control (QC) of participants and genotypes was performed using a standard procedure [27–30]. Briefly, there were no individuals with incorrect sex assignment, excessive heterozygosity, or low call rate (< 98%). There were no incorrect mother-child relationship and cryptic relatedness, as assessed by identity-by-descent (IBD) estimation. Principal component analysis (PCA) anchored with HapMap III reference samples confirmed that all samples taken were from individuals of East Asian descent. At a marker level, SNPs with low call rate (< 95%), low minor allele frequency (MAF; < 0.01), or significant deviation from Hardy-Weinberg equilibrium (*p* < 1 × 10^−5^) were excluded. We excluded non-autosomal chromosomes from the analysis.

Following QC procedures, we conducted genome-wide imputation using the 1000 Genome Phase 3 reference panel [31]. SNP alleles were oriented to the forward strand of the GRCh37/hg19 reference sequence of the human genome. Haplotype phasing and imputation were performed in all maternal and fetal samples using Beagle 5.1 software [32, 33]. After the imputation, we excluded variants with the measurement Dosage R-squared (DR2) < 0.7 or those with low MAF (< 0.01). The sample size was 93, which was equivalent to the minimum target sample size for PGS analysis [27].

### Construction of individual PGSs

A classic clumping and thresholding method was used to derive individual polygenic scores (PGSs) for the target BC-GENIST participants. As a base data, we used GWAS summary statistics (the normalized effect sizes and *p* values) for BP phenotypes (SBP, DBP, MAP, and PP) from Japanese individuals (n > 136,000) from the BioBank Japan (BBJ) [34]. We performed QC relevant to base and target data for the PGS calculation [27]. The reported heritability estimates of SBP, DBP, MAP, and PP were 0.0569, 0.04, 0.0496, and 0.0438, respectively [35]. First, a set of independent variants was extracted using linkage disequilibrium (LD) clumping [30] with the following flags: --clump-p1 1 --clump-p2 1 --clump-r2 0.1 --clump-kb 1000. Next, we selected variants meeting the following *p* value thresholds: 1 × 10^−6^, 1 × 10^−5^, 1 × 10^−4^, and 1 × 10^−3^. The trait variance explained the derived PGSs by calculating the adjusted *R*^2^ attributable to the PGSs was mostly highest at the threshold of 1 × 10^−4^. We derived PGSs by multiplying the dosage of BP-increasing alleles for each variant by the normalized effect size in the GWAS and summing the scores across all the selected variants unless otherwise indicated. The PGSs were scaled to have zero mean and unit variance by *Z* score transformation to compare the effect sizes across the investigated phenotypes.

In order to clarify that the placenta specifically mediates the effect of vasculature-related SNPs among BP SNPs, we constructed a genetic BP-increasing risk score limited to vasculature-related SNPs. Previously, a large-scale of GWAS was performed for SBP, DBP, MAP, and PP, respectively [34], but the associated SNPs were highly overlapping across multiple BP phenotypes. Additional file 1-Table S2 shows 41 SNPs associated with SBP, DBP, MAP, and PP with genome-wide significance. As described in “Variant annotation and relevance to the vascular system” section, 41 SNPs were annotated for their relevance to the vasculature. Then, two distinct genetic risk scores were constructed: one from only “vasculature-related” SNPs and the other from “unlikely related” SNPs. Because the effect size of each SNP varies among the four BP phenotypes, alleles with SBP-increasing effect were counted in an unweighted manner. The reported directions of BP-increasing effects of 41 SNPs were mostly concordant for all BP phenotypes. The effect allele (which denotes the BP-increasing allele) of seven PP-associated SNP presented a DBP-decreasing effect (see gray cells in Additional file 1: Table S2). For these seven discordant SNPs, counting SBP-increasing alleles was rational because the direction was the same among PP, SBP, and MAP.

### Determination of parental origin and computation of individual haplotype allele counts

For a series of genome-wide significant SNPs associated with any BP phenotypes (*p* < 5.0 × 10^−8^) reported by Kanai et al. [34], we estimated mother-offspring allele transmission for each mother-offspring pair after phasing and imputation using Beagle 5.1 software [32, 33]. When either or both mother and fetus were homozygote, the allelic transmission could be unambiguously determined. When both mother and fetus were heterozygote, allelic transmission from mother to child was inferred based on local haplotype sharing, following the method described by Zhang et al. [36]. For each SNP, we calculated three haplotype allele counts: [1] unweighted sum of the number of BP-increasing alleles in maternal transmitted haplotypes, [2] unweighted sum of the number of BP-increasing alleles in maternal non-transmitted haplotypes, and [3] unweighted sum of the number of BP-increasing alleles in paternal transmitted haplotypes.

### Variant annotation and relevance to the vascular system

Genome-wide significant SNPs associated with BP phenotypes [34] were annotated with genes using data obtained on 12/18/2020 from multiple bioinformatic tools such as Variant Effect Predictor (VEP, Ensemble release 102) [37], SNPnexus [38], and post-GWAS [39]. We evaluated all SNPs in LD (*r*^2^ ≥ 0.8) with each variant for evidence of mediation of expression quantitative trait loci (eQTLs) in all 54 tissues using the GTEx database Release V8, to identify affected genes and tissues. GeneHancer database [40] was also used to identify genes potentially affected by SNPs (Additional file 1: Table S2-3).

For the relevance to the vascular system, we assessed all SNPs in LD (*r*^2^ ≥ 0.8) with each variant for evidence of the overlap with ENCODE DNase I regulatory regions and/or Candidate Cis-regulatory elements [38, 41] in blood vessels (umbilical vein endothelial cell [HUVEC]; brain microvascular endothelial cell [HBMEC]; lung microvascular epithelium blood [HMVEC-LBl]; brain vascular smooth muscle cell [HBVSMC]; pulmonary artery fibroblast [HPAF]; aorta fibroblast [AoAF]). The annotated gene relevance to the vasculature (function of the vascular smooth muscle, the vascular endothelial cells, the vascular tone, the vascular assembly, placental vasculature, and HDP; Additional file 1: Table S2-3) was deciphered by a literature review. Among 42 BP-associated SNPs reported [34], rs139293840 (located in *RPTOR*) was excluded for the analysis, as it is monomorphic in non-Asian population and there was no available GTEx data.

### Statistical analyses

R software (version 4.0.3) was used for the statistical analyses. All tests were two-sided. All the variables were scaled by *Z* score transformation. Associations between PGSs and various phenotypes were examined by regression analysis. For the multiplicity adjustments, Bonferroni-adjusted *p* value was noted (Additional file 1: Table S4-10). Since only pre-pregnancy BMI (BMI was log-transformed) among several potential BP confounders (including maternal age, smoking status, and the presence or absence of glucose metabolism impairment) was associated with BP phenotypes, birth weight, and placental weight, the pre-pregnancy BMI was used as a confounder. The birth weight and placental weight used in this study were the variables adjusted for fetal sex, parity, and gestational age using a national reference chart.

Global Validation of Linear Model Assumptions (GVLMA) [42] and the Akaike information criterion (AIC) were used to evaluate whether a simple linear or a quadratic model was appropriate to explain the association between observed maternal BP and birth weight.

Since maternal SBP PGS (exposure) and placental weight (mediator), and placental weight (mediator) and offspring birth weight (outcome) were significantly associated, a causal mediation analysis was performed using the R package “mediation (ver.4.5.0)” [43, 44] to assess the meditation effect of placental weight. Pre-pregnancy BMI was a confounder for placental weight (mediator) and birth weight (outcome). The average causal mediation effects (ACME) were estimated to quantify the mediation effect of placental weight on offspring birth weight. Proportion mediated was derived from the ratio between ACME and total effect. We performed additional mediation analyses using SBP PGS constructed with SNPs not associated with type 2 diabetes mellitus (T2D), hemoglobin A1c (HbA1c), and BMI, as the exposure. Sensitivity analysis for sequential ignorability [43, 44] was performed to show the robustness of the mediator effect to the violations of the assumption, that is, the possible existence of unobserved confounders.

Association between haplotype-based BP-increasing allele counts and placental weight was examined by regression analysis using the polygenic allele counts of maternal transmitted, maternal non-transmitted, and paternal transmitted allele, respectively.

To visualize the relationship between BP-increasing PGS and fetal growth velocity, we stratified the participants according to the tertiles of normalized PGS.

A minimum required sample size of 47 for the linear regression was calculated with an alpha value of 0.05, a power of 0.80, and an *R*^2^ of 0.15 representing the moderate effect size, using the pwr.f2.test function in the pwr package [45].

**Fig. 1:**
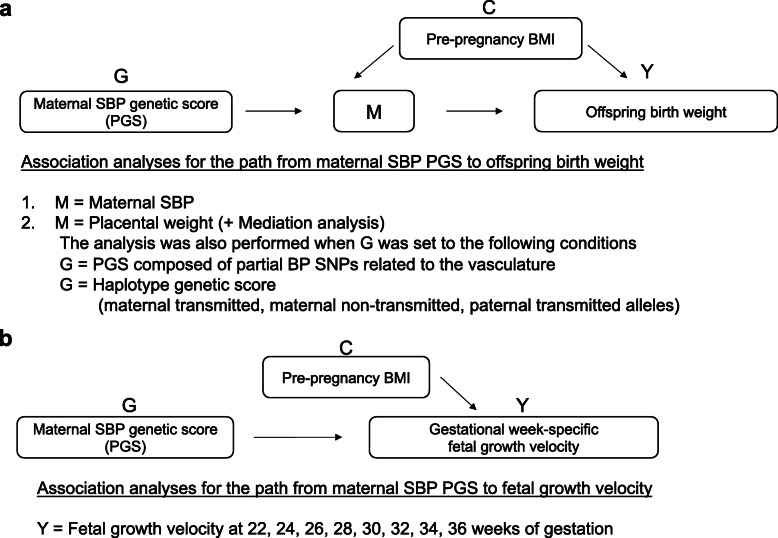
Summary of the associations examined. **a** Schematic representation of the mediated association between the maternal SBP-increasing genetic score and offspring birth weight, and a summary of association analyses. **b** Schematic representation of the association between the maternal SBP-increasing genetic score and fetal growth velocity, and a summary of an association analysis. G, genetic score as an exposure; M, mediator as an intermediate phenotype; Y, outcome; C, confounder; PGS, polygenic score; SBP, systolic blood pressure; BMI, body mass index

All the associations investigated in this study are schematically represented in Fig. 1.

## Results

### Study participants

The basic characteristics of the study participants are described in Additional file 1: Table S1, and no differences were observed between the BC-GENIST and the TMDU pregnant women cohorts in terms of maternal characteristics and birth outcomes. Briefly, less than 9% were overweight/obese while more than 17% were underweight (Additional file 1: Table S1). As stated in the “Methods” section, chronic hypertension and HDP were excluded in this study.

### Association of BP-PGS with offspring birth weight and placental weight

High maternal PGS for each BP phenotype (SBP, DBP, MAP, or PP; exposure) was negatively associated with birth weight (outcome) (Additional file 1: Tables S4-7). Among four BP phenotypes, SBP-PGS (111 SNPs, GWAS *p* value threshold = 1.00 × 10^−5^) was inversely associated with birth weight with the highest significance (estimated regression coefficient [Est.; 95% CI] = − 0.340 [− 0.509, − 0.171], *p* = 1.29 × 10^−4^). Although association between maternal SBP-PGS and placental weight (potential mediator) was significant (Est. [95% CI] = − 0.396 [− 0.556, − 0.236], *p* = 3.98 × 10^−6^), there was no correlation between SBP-PGS and observational SBP traits (*p* = 0.736; Additional file 1: Tables S4).

### Mediation models of the relationship between maternal SBP-PGS and offspring birth weight

As for potential mediator-outcome association, the linear regression model of birth weight (outcome) on placental weight (potential mediator) was well fitted and the Est. was 0.661 [95% CI 0.50, 0.82] (*p* value =1.7 × 10^−12^). In contrast, linear regression models of birth weight on SBP trait were not fitted well (Additional file 2: Figures S1-S3). In addition, placental weight was never negatively associated with BP traits (Additional file 1: Table S8).

**Fig. 2:**
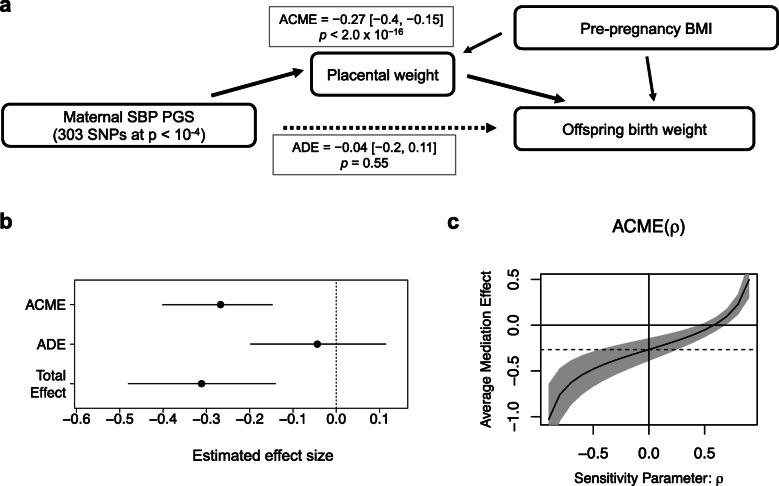
Causal mediation analysis. **a**, **b** Mediation model of the relationship between maternal SBP-increasing PGS (exposure), placental weight (mediator), and birth weight (outcome). Placental weight has a significant average causal mediation effect (ACME) for decreasing birth weight by maternal SBP PGS (effect = − 0.27 [− 0.4, − 0.15], *p* < 2.0 × 10^−16^), whereas the average direct effect (ADE) is not significant (effect = − 0.04 [− 0.2, 0.11], *p* = 0.55). **c** The sensitivity parameter *ρ* denotes the correlation coefficient between the residuals of the mediator and outcome regressions [44]. A sensitivity analysis performed by examining how the estimated ACME changes while the value of *ρ* varies shows that ACME estimates are negative and not altered for a wide range of ρ values below 0.6

Causal mediation analysis with the quasi-Bayesian Monte Carlo method estimated that 86% of the effect of maternal SBP-increasing PGS on birth weight was mediated by placental weight (Fig. 2a, Additional file 1, Table S9). The average causal mediation effects (ACME) was − 0.27 [95% CI − 0.40, − 0.15] (*p* value < 2.0 × 10^−16^) whereas the average direct effects (ADE) was − 0.049 [− 0.21, 0.12] (*p* value = 0.54; Additional file 1: Table S9 and Figure 2b). Even after excluding pleiotropic SNPs associated with either BMI, T2D, or HbA1c at *p* value thresholds of 1 × 10^−8^, 1 × 10^−6^, or 1 × 10^−4^, the mediation effect estimates of placental weight were not changed (Additional file 1: Table S9). To assess the extent of estimate robustness to plausible violation of assumption on unmeasured confounder, we performed a sensitivity analysis for sequential ignorability [43, 44] (Fig. 2c). The ACME estimates were not altered much for a wide range of *ρ* values below 0.6, suggesting that the mediation effects are largely robust to potential violation for sequential ignorability assumption which includes the ignorability assumption of unmeasured variables that confound the relationship between the mediator and the outcome.

Causal mediation analysis for other significantly birth weight-associated PGS, SBP-PGS (111 SNPs, *p* value thresholds = 1 × 10^−5^), and PP-PGS (29 SNPs, *p* value thresholds = 1 × 10^−6^) estimated that 70% and 72%, respectively, of the effect of maternal BP-increasing PGS on birth weight was mediated by placental weight (Additional file 2: Figure S4).

### The placenta mediates the effect of vasculature-related BP-increasing SNPs on offspring birth weight

**Fig. 3:**
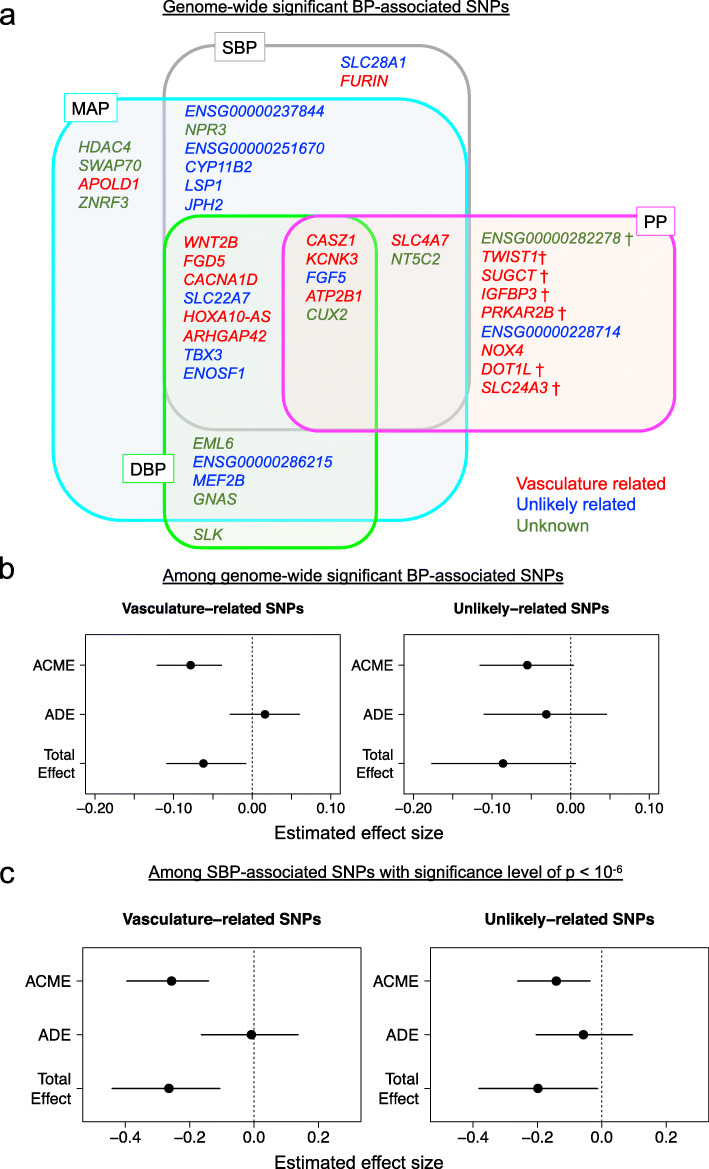
Placental weight mediates the effects of "vasculature-related" blood pressure SNPs on birth weight. **a** Venn diagram of 41 genome-wide significant single nucleotide polymorphisms (SNPs) for BP phenotypes. There is a considerable overlap of association across four BP phenotypes, with a consistent direction of the BP increasing effect, except for seven loci that present opposite (BP decreasing) or unclear effect directions for DBP (indicated by †). SNPs are classified into “vasculature-related” (red), “unlikely related” (blue), and “unknown” (green) groups, respectively, as described in the results and Additional File 1: Table S2. **b** Mediation analysis of the models of either the PGS with vasculature-related BP SNPs (left) and unlikely related BP SNPs (right) as an exposure variable. Maternal PGS of “vasculature-related” SNPs shows a birth weight reducing effect (total effect = − 0.06 [− 0.11, − 0.01], *p* = 0.032) and placental weight has a significant mediation effect (ACME = − 0.08 [− 0.12, − 0.04], *p* < 2.0 × 10^−16^). Maternal PGS of “unlikely-related” SNPs do not significantly reduce birth weight (total effect = − 0.09 [− 0.18, 0.01], *p* = 0.082). **c** Mediation analysis of the models of either the SBP-increasing PGS with “vasculature-related” SNPs (left) and with “unlikely related” SNPs (right) as an exposure variable. Of the total effect of “vasculature-related” PGS on birth weight (total effect = − 0.26 [− 0.44, − 0.11], *p* = 0.002), the mediated effect by placental weight accounts for the most part (ACME = − 0.26 [− 0.40, − 0.14], *p* < 2.0 × 10^−16^). Smaller portion (around 69%) of the total effect of “unlikely related” PGS (total effect = − 0.20 [− 0.38, − 0.01], *p* = 0.034) is mediated by placental weight (ACME = − 0.14 [− 0.26, − 0.04], *p* = 0.01)

To focus on the relevance to the vasculature, we constructed a maternal genetic risk score limited to vasculature-related SNPs from 41 SNPs associated with SBP, DBP, MAP, and PP identified by Kanai et al. [34] with genome-wide significance (Additional file 1: Table S2 and Fig. 3a). Of the 41 SNPs, 18 were in the “vasculature-related,” 12 in the “unlikely related,” and 11 in the “unknown” classification (Fig. 3a). Whereas “vasculature-related” genetic risk score was inversely associated with birth weight (Est. (change in birth weight *z* score per allele) = − 0.06 [− 0.12, − 0.01]; *p* = 2.5 × 10^−2^) and placental weight (Est. = − 0.11 [− 0.16, − 0.06]; *p* = 1.7 × 10^−5^), “unlikely-related” genetic risk score was associated neither with birth weight (*p* = 0.087), nor with placental weight (*p* = 0.083). Causal mediation analysis estimated nearly 100% of the effect of “vasculature-related” genetic score on birth weight was mediated by placental weight (Fig. 3b).

To confirm that the association of “vasculature-related” genetic risk score with placental weight was not due to the larger number of SNPs, we also performed a similar subdivided PGS analysis, using the top 67 SBP-SNPs (SBP association GWAS *p* value < 1 × 10^-6^; Additional file 1: Table S3). Of these, 21 were in the “vasculature related,” 30 in the “unlikely related,” and 16 in the “unknown” classification. The “vasculature-related” PGS was more strongly associated with birth weight than the “unlikely related” PGS and was associated with birth weight (Est. (change in birth weight *z* score per 1 SD increase of PGS) = − 0.26 [− 0.44, − 0.09]; *p* = 3.6 × 10^−3^) and placental weight (Est. = − 0.39 [− 0.55, − 0.23]; *p* = 5.7 × 10^−6^). Causal mediation analysis estimated 96% of the “vasculature-related” PGS effect was mediated by the placental weight (Fig. 3c).

### Haplotype-based genetic score analysis confirmed the role of maternal alleles

To determine the relative contributions of maternal and fetal genotypes to the relationship between BP-increasing alleles and birth weight/placental weight, we estimated mother-offspring allele transmission based on local haplotype sharing [36] for the BP-associated SNPs with genome-wide significance [34] (Fig. 3a). Both the transmitted and non-transmitted maternal haplotype genetic scores (allele counts) were inversely correlated with birth weight (Est. = − 0.07 [− 0.13, − 0.011], *p* = 2.1 × 10^−2^; Est. = − 0.092 [− 0.16, − 0.026], *p* = 6.8 × 10^−3^, respectively). On the contrary, we did not observe any correlation between paternally inherited haplotype genetic score and birth weight (*p* = 0.25; Additional file 1: Table S10). Similarly, both transmitted and non-transmitted maternal genetic scores were inversely associated with placental weight (Est. = − 0.14 [− 0.19, − 0.082], *p* = 2.3 × 10^−6^; Est. = − 0.094 [− 0.16, − 0.029], *p* = 4.8 × 10^−3^, respectively), whereas there was no association with paternally inherited haplotype genetic score (*p* = 0.25; Additional file 1: Table S10).

### The inverse association between maternal SBP PGS and fetal growth velocity only became apparent in late gestation

**Fig. 4:**
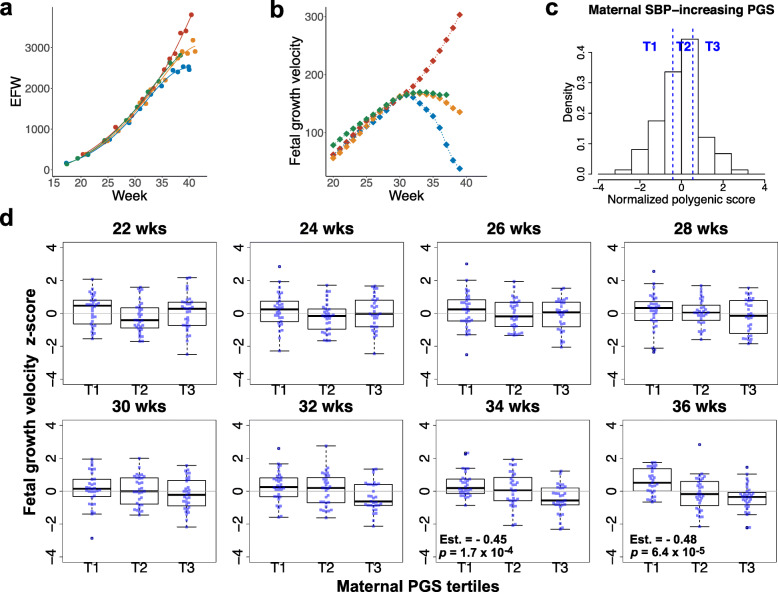
Effects of maternal SBP-increasing PGS on gestational week-specific fetal growth velocity. **a** Estimation of gestational week-specific estimated fetal weight (EFW) for individual fetus. Longitudinal ultrasound scan data at prenatal checkups are collected for individual fetus (colored circles) and the weekly values (continuous values) of EFW was estimated by smoothing splines interpolation. Data examples for four fetuses are shown (each fetus with a different color). **b** Gestational week-specific fetal growth velocity was calculated as the difference between two consecutive weekly EFW values. Fetuses are shown the same color as in **a**. **c** Distribution of normalized maternal SBP-increasing polygenic score (PGS) constituted of a set of SNPs exceeding a GWAS association *p* value of 1.0 × 10^−4^ and the stratification according to the tertiles. **d** Fetal growth velocity across maternal PGS tertiles. Each subject (*n* = 93) is shown in the box-and-whisker plots, with the boxes indicating the median and interquartile range and the whiskers denoting the range. Regression coefficient (Est.), standing for the change per strata increase, and *p* value are shown when *p* value is below the Bonferroni-corrected threshold for 8 tests (*p* = 6.3 × 10^−3^)

We collected longitudinal ultrasound measurements of estimated fetal weight (EFW) and estimated the weekly values of EFW by smoothing splines interpolation (Fig. 4a). We then differentiated them to calculate fetal growth velocity every week (individual changes in EFW per week; Fig. 4b) and normalized them by *z* score transformation. We stratified the participants according to the tertiles of maternal SBP-increasing PGS consisting of SNPs meeting the *p* value threshold of 1.0 × 10^−4^ (Fig. 4c). Linear regression analysis was performed every two weeks from 22 to 36 weeks, and the results showed that the inverse association between maternal SBP-increasing PGS and fetal growth velocity became progressively apparent towards 36 weeks (Fig. 4d).

## Discussion

This is the first study to show that placental weight mediates the effect of maternal SBP-increasing PGS on lowering birth weight. While we found a notable inverse association between maternal genetic score and placental weight, observational BP traits were not associated with BP genetic score. In addition, placental weight well correlated with birth weight, whereas no correlation was observed between observational BP and birth weight. PGS constituted of BP SNPs only related to the vascular system also had a reducing effect on birth weight, and this effect was mediated by the placental weight. Additionally, we put in evidence that both maternal transmitted and non-transmitted alleles of BP-increasing SNPs, but not paternal transmitted alleles, affect birth weight. The association between maternal SBP PGS and deceleration of fetal growth velocity only appeared later in gestation, consistent with a placental mediation of the effect of maternal hypertension genetic risk on birth weight.

Previous MR studies of the European ancestry population have consistently reported an inverse association between maternal SBP-increasing PGS and birth weight [7, 8, 10]. In the present study based on Japanese pregnant women, we replicated this association with linear regression analyses, although SNPs constituting PGS for the Japanese population differed from those for the European population.

Using MR analysis, Tyrrell et al. estimated that every 10 mmHg increase of the maternal SBP is genetically associated with a 208-g decrease in birth weight [8]. However, they observed that a 10-mmHg increase in measured maternal SBP was associated with a 24-g increase in birth weight [8]. That is, the direction of the observed association was opposite to the estimated genetic association. This was inconsistent with the assumption that SBP traits mediate the effect of maternal SBP genetic score on birth weight, but it was thought to be attributed to confounding, such as BMI. Since then, this issue has not been fully explained. Our results indicate that it is unlikely that maternal BP traits mediate the effect of maternal BP PGS on birth weight, even when pre-pregnancy BMI is included as a confounder in the analysis. In contrast, the present study found that maternal SBP increasing genetic score was associated with reducing placental weight, and placental weight correlated with birth weight. A mediation analysis showed that more than 70% of the effect of maternal SBP increasing PGS on birth weight was mediated by placental weight. The results of the mediation analysis were not altered by the exclusion of SNPs associated with BMI, HbA1c, and T2D among the SBP-associated SNPs. Furthermore, the result of our sensitivity analysis for sequential ignorability [43, 44] also showed that even if there were unmeasured confounders, they would not affect the conclusion that the placenta mediates the effect of SBP PGS on birth weight.

Genes associated with hypertension have been identified by recent GWAS [16–18], and beyond genetic variants that work in the kidney and adrenal glands, many SNPs have been shown to be related to vascular function, vascular development, angiogenesis, and remodeling. This prompted us to subdivide BP SNPs into either vasculature-related or unlikely-related groups and examine the association of their genetic scores with birth weight. We observed that the genetic score of SNPs related to the vascular system presented a higher association with birth weight, and this was mostly mediated by placental weight.

To analyze the maternal genetic score as a proxy for the intrauterine environment, Beaumont, Warrington, and their colleague developed a method of analysis that distinguishes it from the effects of genetic factors shared by the fetus [7, 10]. They showed that the effect of the SBP-increasing alleles on lowering birth weight originated from maternal alleles. In the present study, we used a method [36] to calculate genetic scores separately for maternal transmitted, maternal non-transmitted, and paternal transmitted alleles, and confirmed that the effects of BP-increasing alleles on birth weight and placental weight were only observed for maternal alleles. The results are in agreement with previous studies [7, 10].

Fetal growth restriction (FGR) is characterized as a growth deceleration from a certain point in the gestation period, resulting in fetus pathologically small for the gestation period (SGA), which is different from constitutionally SGA [4, 46]. As most FGR are thought to arise from placental dysfunction, which matures in the 3rd trimester and slows down its growth after around 30 weeks, it appears reasonable that nearly 80% of FGR are late-onset (≥ 32 weeks) [13]. When and how the fetal growth velocity changes are important information. We previously calculated the EFW increase per week (differential value) every week, which is called fetal growth velocity, for individual fetuses and analyzed the trajectories of the velocity. We have shown that the heterogeneity of growth velocity trajectories is most pronounced after 30 weeks of gestation [20]. Individual trajectories analysis highlighted the continuity of the degree of growth restriction, and ≥ 10% of the fetuses showed a third-trimester growth deceleration. Considering the time sequence of events, maternal PGS should be associated with fetal growth velocity in late gestation where placental maturation is proceeding. As expected, the inverse association between maternal SBP-increasing PGS and fetal growth velocity became progressively apparent towards 36 weeks. This result, although indirect, further supports a placental mediation of the effects of maternal BP genetic score on birth weight.

The NICHD Fetal Growth Study, which explored the association between maternal SBP (or DBP)-increasing PGS and EFW for each trimester, has been recently reported [47]. A unique feature of the NICHD study is that ethnically different populations (Hispanic, non-Hispanic White, non-Hispanic Black, and Asian) were included in the analysis. The authors referred to the GWAS summary statistics of European ancestry to construct PGS and separately applied it to all distinct target populations. However, it is well known that PGS analyses using the summary statics of ethnically different population from the target population are not reliable [27, 48]. In addition, since the frequency of ultrasound measurements was not very high, the estimation of each individual gestational week-specific EFW might have not been accurate. In our analysis, the study target population was limited to Japanese, and PGS analysis was performed using summary statistics of Japanese GWAS to obtain reliable results. In addition, we were able to collect a much larger number of ultrasound measurements for every fetus so that we could obtain weekly EFW values and their differentiated value, fetal growth velocity [20]. We focused our analysis on the association between the gestational week-specific fetal growth velocity and maternal SBP PGS, and we found that this association only became apparent late in gestation.

Up to now, maternal genetic scores associated with SBP, DBP, BMI, and T2D or birth weight-associated SNPs were used as a proxy for the intrauterine environment to validate Developmental Origin of Health and Disease (DOHaD) hypothesis [7–10]. Recent MR studies have concluded that the intrauterine environment is unlikely to be a major determinant of adverse cardiometabolic outcomes in offspring [10]. However, our study reveals a significant association between placental weight and maternal PGS composed of partial BP SNPs related to the vasculature. This result suggests that it is possible to design a maternal genetic score representing the intrauterine environment much better. A large placental weight GWAS using mother and child genome will be needed to investigate the association between the maternal genetic score related to placental weight and the offspring’s birth weight and cardiometabolic traits in adulthood. Alternatively, since placental function may not necessarily correlate with placental weight, we need to devise a way to evaluate the genetic score that more directly reflects the intrauterine environment. It will also be interesting to see what the impact on the cardiometabolic outcome of the offspring will be in MR approach if maternal BP SNPs are limited to the vasculature and/or placenta-related SNPs. In any case, we think more analyses need to be performed before intrauterine programming is ruled out.

The main strength of our study was using the cohort of mother-offspring pair cohort that contains unique phenotype data for time-series fetal growth velocity and placental weight as well as genotype. In addition, birth weight and placental weight were ideally adjusted for gestational age, fetal sex, and maternal parity to eliminate well-known confounding factors. Pre-pregnancy BMI was also considered as a confounding factor. Using causal mediation analysis, we found for the first time that the effects of maternal SBP PGS on birth weight are mediated by placental weight. However, some limitations should be noted. First, the sample size of the cohort with available genotype was small, around 100, which is the minimum target sample size for PGS analysis [27]. The association between SBP-PGS and placenta was much stronger than the association between typical PGS and the corresponding traits; the variance of placenta trait explained by the PGS, represented by adjusted *R*^2^, exceeded 0.2; therefore, we were able to perform statistically reliable association analysis with this sample size. Nevertheless, the results of this study will need to be replicated in another cohort possessing complete data of fetal growth velocity and placental weight. Second, we classified PGSs based on literature and open-source data on gene activities and chromatin status in vascular tissues for the genes affected by SNPs. Although these methods were commonly used in previous studies [16–18], some information is not yet complete and will improve as more data accumulate.

## Conclusion

In the present study, we show that maternal hypertension genes are strongly associated with placental growth and that maternal genetic risk of hypertension inhibits fetal growth through the intrauterine environment established by the placenta. Our finding is highly important for the perinatal management of FGR. Given that many FGRs are late-onset and difficult to diagnose [4, 11, 13], maternal SBP-PGS will be useful to the screening for late-onset FGR high-risk groups, regardless of the presence or absence of maternal hypertension. It cannot be dismissed that suboptimal intrauterine environment due to reduced placenta growth could generate future hypertension in LBW offspring. This study reconciles the long-held debate on the prenatal origin of diseases, adverse intrauterine environment or genetic effects, and will contribute to the prevention of hypertension and cardiovascular diseases.

## Supplementary Information

**Additional file 1: Table S1.** Characteristics of participants included in this study. **Table S2.** Genome-wide significant loci for blood pressure phenotypes and classification based on vasculature relevance. **Table S3.** Variants constituting SBP-increasing PGS at *p* value threshold of 1 x 10^−6^ and classification based on vasculature relevance. **Table S4.** Linear regression analyses for maternal SBP-increasing PGS predicting various traits including birth weight. **Table S5.** Linear regression analyses for maternal DBP-increasing PGS predicting various traits including birth weight. **Table S6.** Linear regression analyses for maternal MAP-increasing PGS predicting various traits including birth weight. **Table S7.** Linear regression analyses for maternal PP-increasing PGS predicting various traits including birth weight. **Table S8.** Linear regression analyses for maternal BP traits predicting placental weight. **Table S9.** Sensitivity analysis for estimates of the causal mediation analyses by excluding pleotropic SNPs from exposure PGS.**Table S10.** Linear regression analyses for BP-increasing allele predicting birth weight or placental weight.

**Additional file 2: Figure S1.** Relationship between maternal BP in early gestation and offspring birth weight. **Figure S2.** Relationship between maternal BP in mid gestation and offspring birth weight. **Figure S3.** Relationship between maternal BP in late gestation and offspring birth weight. **Figure S4.** Estimates of mediation analyses with different PGS (exposure variable).

## Data Availability

The datasets used and/or analyzed during the current study are available from the corresponding author on reasonable request.

## Abbreviations

ACME: Average causal mediation effects
ADE: Average direct effects
AIC: Akaike information criterion
BBJ: BioBank Japan
BC-GENIST: Birth Cohort Gene and ENvironment Interaction Study of TMDU
BP: Blood pressure
CI: Confidence interval
DBP: Diastolic blood pressure
DOHaD: Developmental Origin of Health and Disease
EFW: Estimated fetal weight
eQTL: Expression quantitative trait loci
FGR: Fetal growth restriction
GVLMA: Global Validation of Linear Model Assumptions
GWAS: Genome wide association study
HbA1c: Hemoglobin A1c
HDP: Hypertensive disorder of pregnancy
IBD: Identity-by-descent
LBW: Low birth weight
LD: Linkage disequilibrium
MAF: Minor allele frequency
MAP: Mean arterial pressure
MR: Mendelian randomization
PCA: Principal component analysis
PGS: Polygenic score
PP: Pulse pressure
QC: Quality control
SBP: Systolic blood pressure
SGA: Small for gestational age
SNP: Single nucleotide polymorphism
T2D: Type 2 diabetes mellitus
TMDU: Tokyo Medical and Dental University
VEP: Variant Effect Predictor
