# Supplementary material for: Placenta mediates the effect of maternal hypertension polygenic score on offspring birth weight: a study of birth cohort with fetal growth velocity data
Source: BMC Med. 2021 Nov 4;19:260. doi: 10.1186/s12916-021-02131-0 (PMC8567693; doi:10.1186/s12916-021-02131-0)
Supplement: Supplementary file 2 — Additional file 2: Figure S1. Relationship between maternal BP in early gestation and offspring birth weight. Figure S2. Relationship between maternal BP in mid gestation and offspring birth weight. Figure S3. Relationship between maternal BP in late gestation and offspring birth weight. Figure S4. Estimates of mediation analyses with different PGS (exposure variable). [file 12916_2021_2131_MOESM2_ESM.pptx]

## Slide 1
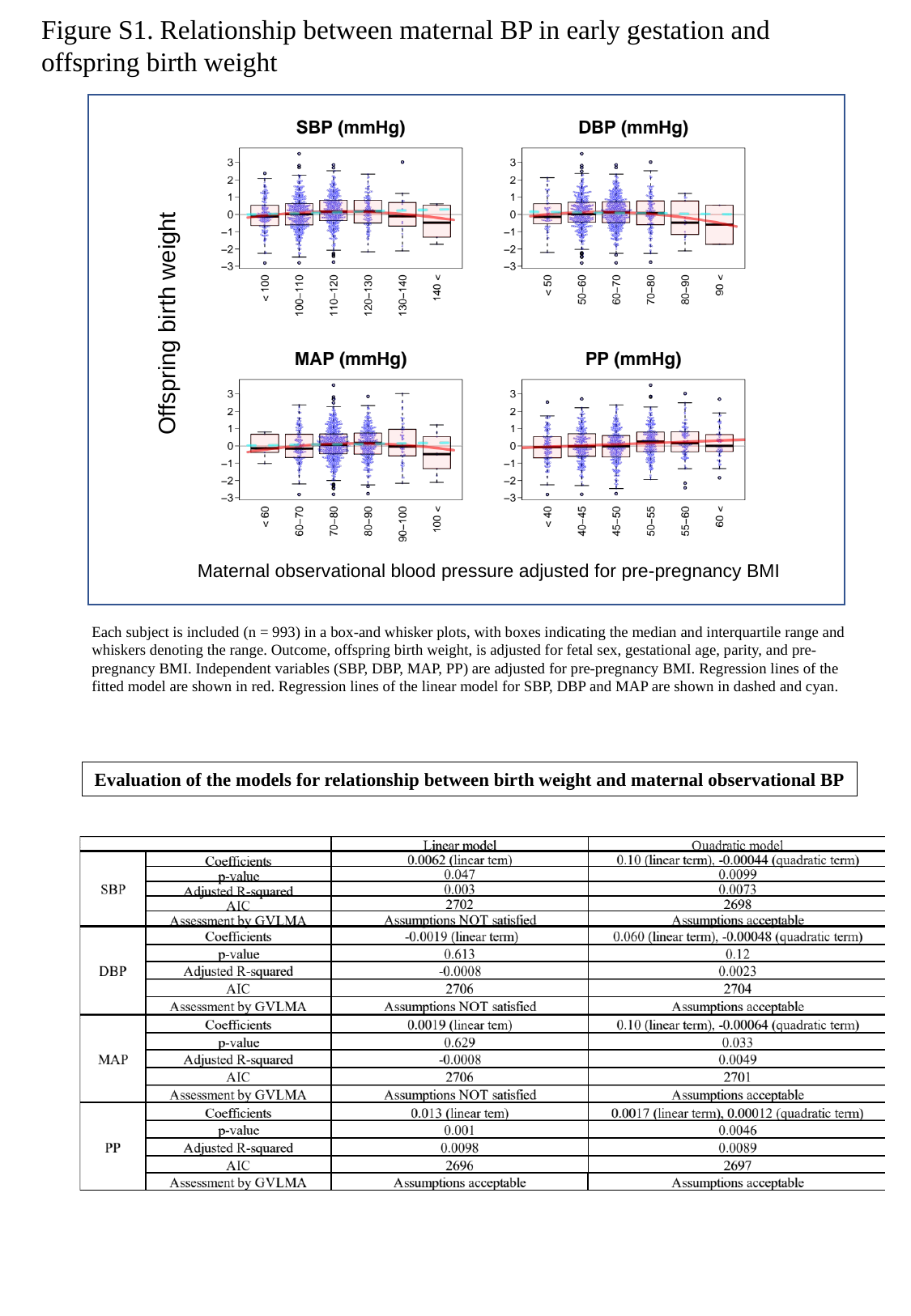

Figure S1. Relationship between maternal BP in early gestation and offspring birth weight
Offspring birth weight
Maternal observational blood pressure adjusted for pre-pregnancy BMI
Each subject is included (n = 993) in a box-and whisker plots, with boxes indicating the median and interquartile range and whiskers denoting the range. Outcome, offspring birth weight, is adjusted for fetal sex, gestational age, parity, and pre-pregnancy BMI. Independent variables (SBP, DBP, MAP, PP) are adjusted for pre-pregnancy BMI. Regression lines of the fitted model are shown in red. Regression lines of the linear model for SBP, DBP and MAP are shown in dashed and cyan.
Evaluation of the models for relationship between birth weight and maternal observational BP

## Slide 2
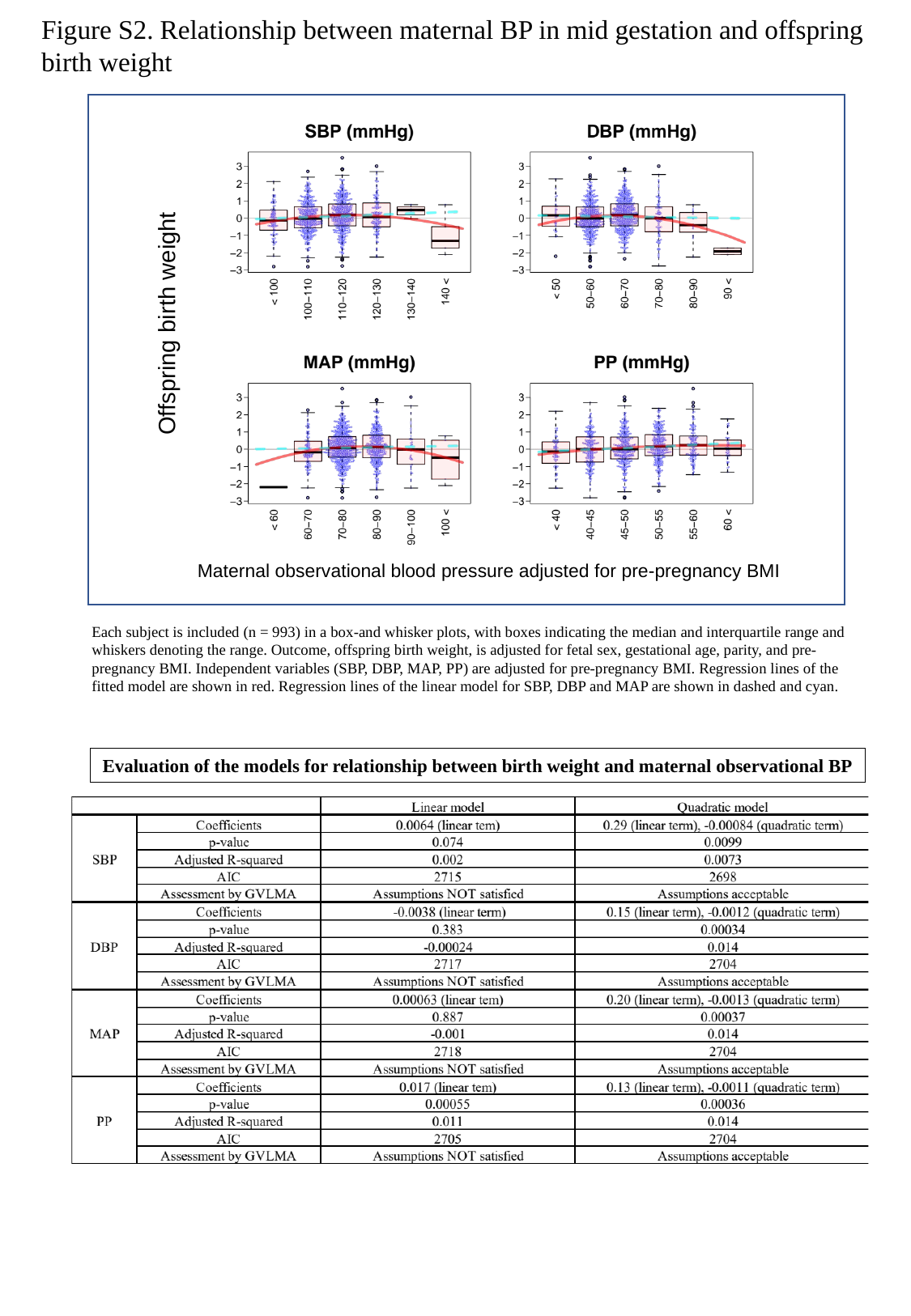

Figure S2. Relationship between maternal BP in mid gestation and offspring birth weight
Offspring birth weight
Maternal observational blood pressure adjusted for pre-pregnancy BMI
Each subject is included (n = 993) in a box-and whisker plots, with boxes indicating the median and interquartile range and whiskers denoting the range. Outcome, offspring birth weight, is adjusted for fetal sex, gestational age, parity, and pre-pregnancy BMI. Independent variables (SBP, DBP, MAP, PP) are adjusted for pre-pregnancy BMI. Regression lines of the fitted model are shown in red. Regression lines of the linear model for SBP, DBP and MAP are shown in dashed and cyan.
Evaluation of the models for relationship between birth weight and maternal observational BP

## Slide 3
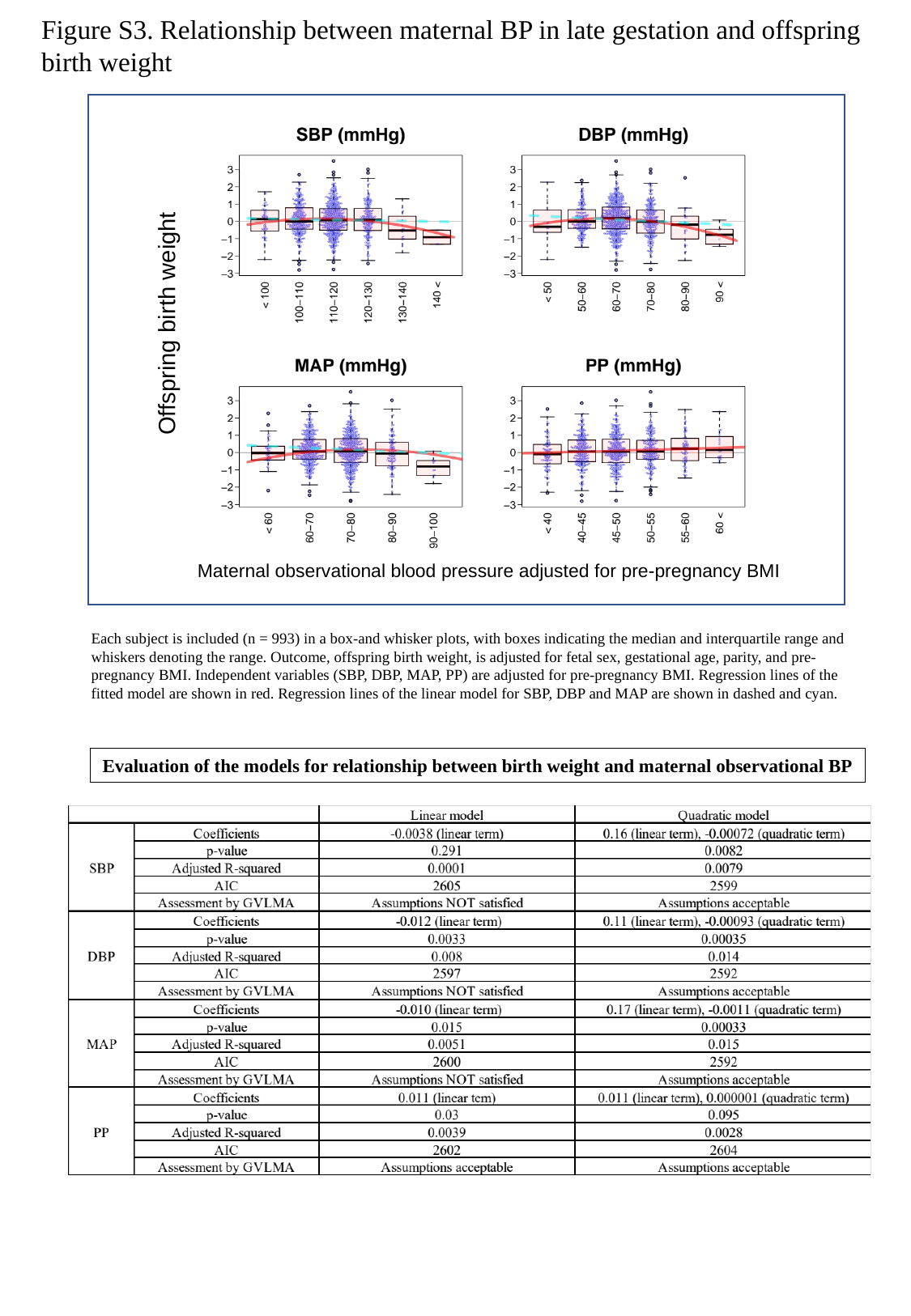

Figure S3. Relationship between maternal BP in late gestation and offspring birth weight
Offspring birth weight
Maternal observational blood pressure adjusted for pre-pregnancy BMI
Each subject is included (n = 993) in a box-and whisker plots, with boxes indicating the median and interquartile range and whiskers denoting the range. Outcome, offspring birth weight, is adjusted for fetal sex, gestational age, parity, and pre-pregnancy BMI. Independent variables (SBP, DBP, MAP, PP) are adjusted for pre-pregnancy BMI. Regression lines of the fitted model are shown in red. Regression lines of the linear model for SBP, DBP and MAP are shown in dashed and cyan.
Evaluation of the models for relationship between birth weight and maternal observational BP

## Slide 4
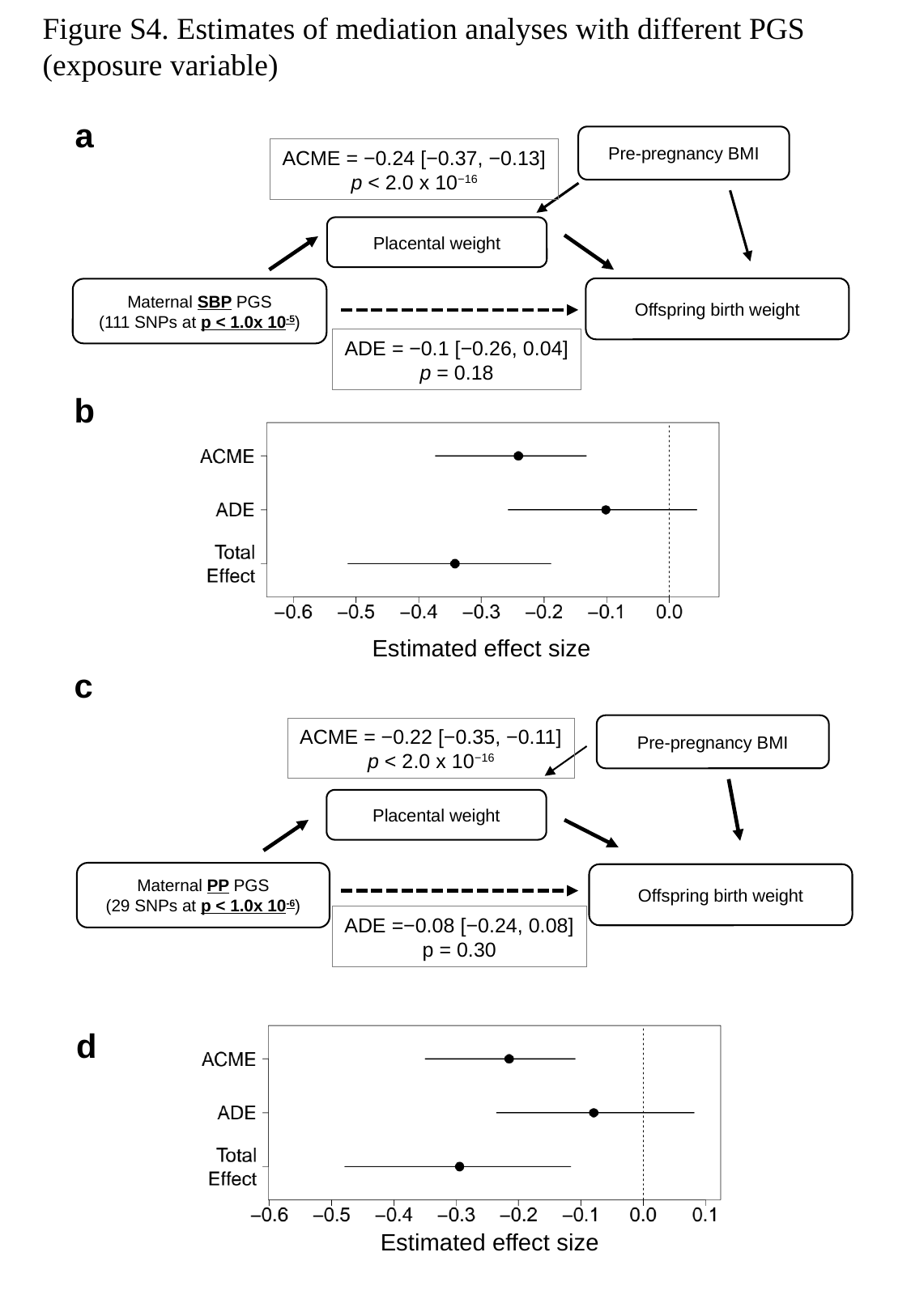

Figure S4. Estimates of mediation analyses with different PGS (exposure variable)
a
Pre-pregnancy BMI
ACME = −0.24 [−0.37, −0.13]
p < 2.0 x 10^−16^
Placental weight
Offspring birth weight
Maternal SBP PGS
(111 SNPs at p < 1.0x 10^-5^)
ADE = −0.1 [−0.26, 0.04]
p = 0.18
b
Estimated effect size
c
Pre-pregnancy BMI
ACME = −0.22 [−0.35, −0.11]
p < 2.0 x 10^−16^
Placental weight
Maternal PP PGS
(29 SNPs at p < 1.0x 10^-6^)
Offspring birth weight
ADE =−0.08 [−0.24, 0.08]
p = 0.30
d
Estimated effect size

## Slide 5
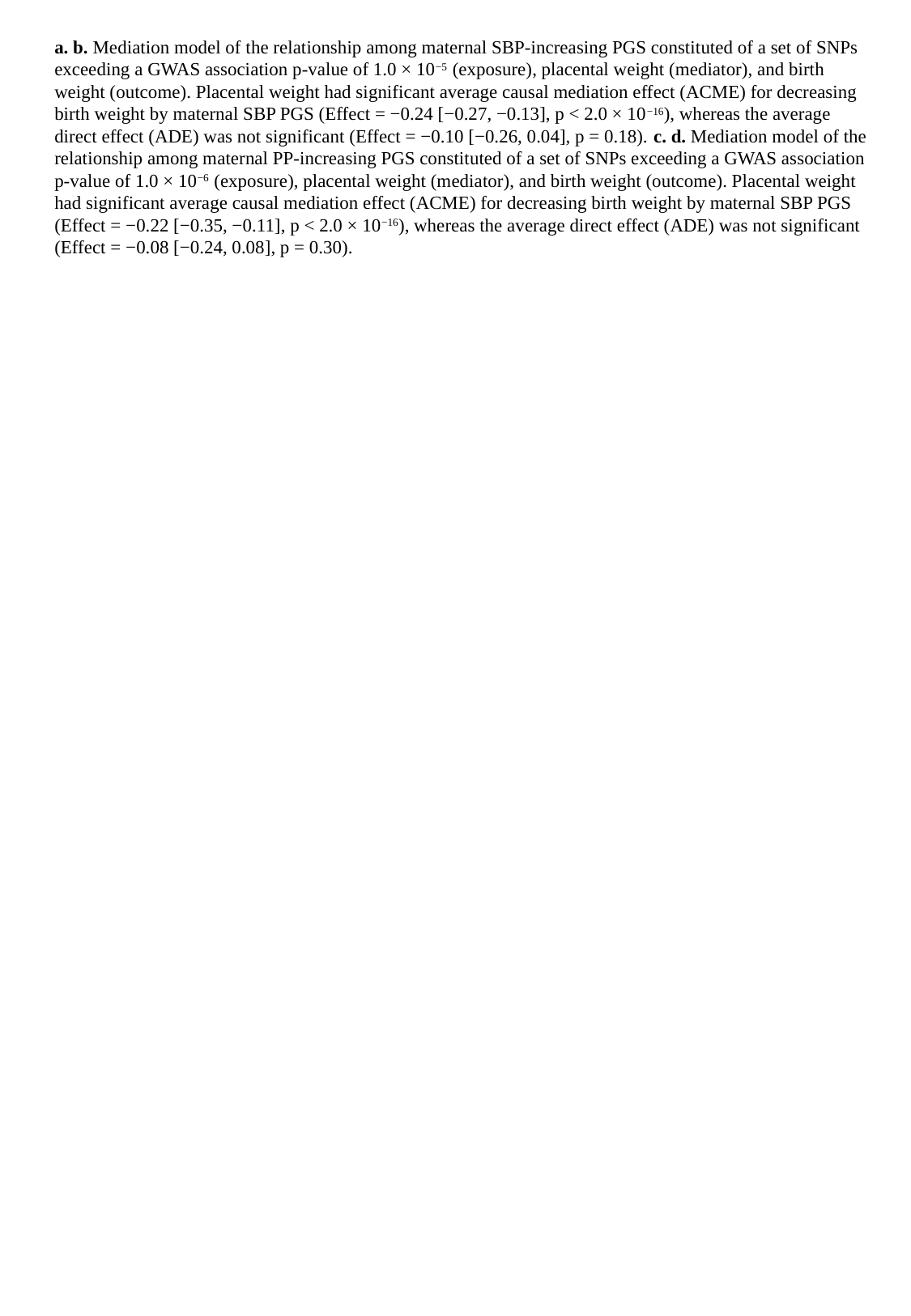

a. b. Mediation model of the relationship among maternal SBP-increasing PGS constituted of a set of SNPs exceeding a GWAS association p-value of 1.0 × 10^−5^ (exposure), placental weight (mediator), and birth weight (outcome). Placental weight had significant average causal mediation effect (ACME) for decreasing birth weight by maternal SBP PGS (Effect = −0.24 [−0.27, −0.13], p < 2.0 × 10^−16^), whereas the average direct effect (ADE) was not significant (Effect = −0.10 [−0.26, 0.04], p = 0.18). c. d. Mediation model of the relationship among maternal PP-increasing PGS constituted of a set of SNPs exceeding a GWAS association p-value of 1.0 × 10^−6^ (exposure), placental weight (mediator), and birth weight (outcome). Placental weight had significant average causal mediation effect (ACME) for decreasing birth weight by maternal SBP PGS (Effect = −0.22 [−0.35, −0.11], p < 2.0 × 10^−16^), whereas the average direct effect (ADE) was not significant (Effect = −0.08 [−0.24, 0.08], p = 0.30).
